# Real-world Benefits of Diabetes Management App Use and Self-monitoring of Blood Glucose on Glycemic Control: Retrospective Analyses

**DOI:** 10.2196/31764

**Published:** 2022-06-15

**Authors:** Ya-Ting Chang, Yu-Zhen Tu, Hung-Yi Chiou, Ken Lai, Neng Chun Yu

**Affiliations:** 1 H2 Inc Taipei Taiwan; 2 Neng Chun Diabetes Clinic Medical & Health in Lotung Yilan Taiwan

**Keywords:** diabetes care, digital intervention, mobile app, real-world data, glycemic control, mobile health, digital therapeutics, diabetes, therapy, app

## Abstract

**Background:**

Among self-care measures, the self-monitoring of blood glucose (SMBG) is a critical component for checking blood glucose levels. In addition, there is growing evidence suggesting that digital technologies are being adopted as an additional method for health care systems to increase patient contact. However, for patients with non–insulin-treated diabetes mellitus type 2 (DMT2), the value of SMBG was inconsistent among studies, and the evidence for digital technologies from real-world clinical practice is still limited.

**Objective:**

Our study aimed to assess patients with non–insulin-treated DMT2 who were receiving care from a single clinic and analyze whether the use of a diabetes management app and SMBG behavior would affect glycemic control in a real-world clinical setting.

**Methods:**

We collaborated with a large clinic focused on diabetes care in Taiwan that had been using the Health2Sync mobile app and web-based Patient Management Platform to collect the data. The patients were divided into 2 groups (app-engaged-user group and only-data-uploader group) according to different activities in the app, and blood glucose was recorded every month from 1 to 6 months after registration in the app. A sample of 420 patients was included in the analysis, and a linear mixed model was built to investigate which factors affected the patients’ blood glucose percentage change.

**Results:**

Using the mixed model coefficient estimates, we found that the percentage change was significantly negative when the only-data-uploader group was set as the baseline (*t*=–3.873, *df*=1.81 × 10^4^; *P*<.001 for the patients of the app-engaged-user group). We found that for patients with shorter diabetes duration, their blood glucose decreased more than patients with longer diabetes duration (*t*=2.823, *df*=1.71 × 10^4^; *P*=.005 for the number of years of diabetes duration). In addition, we found that for younger patients, their blood glucose decreased more than older patients (*t*=2.652, *df*=1.71 × 10^4^; *P*=.008 for the age of the patients). Furthermore, the patients with an education level of junior high school or lower saw a significantly greater decrease in blood glucose percentage change than the patients with an education level of senior high school or higher (*t*=4.996, *df*=1.72 × 10^4^; *P*<.001 for the patients with an education level of senior high school or higher). We also found that the count of blood glucose measured enlarged the decrease along the interaction months (*t*=–8.266, *df*=1.97 × 10^4^; *P*<.001 for the nth month × the count of blood glucose in the nth month). Lastly, the gender of the patients did not significantly affect the percentage change (*t*=0.534, *df*=1.74 × 10^4^; *P*=.59 for female patients).

**Conclusions:**

Our analysis showed the following: the blood glucose percentage change of the patients in the app-engaged-user group dropped more than that in the only-data-uploader group; shorter diabetes duration is associated with a steeper decrease in the patients’ blood glucose percentage change; the percentage decrease in blood glucose change in younger patients is greater than older patients; the blood glucose percentage change of the patients with an education level of junior high school or lower dropped more than those with an education level of senior high school or higher; and the more frequently the patients test SMBG each month, the greater the decrease in the patients’ blood glucose percentage. Further studies can be performed to consider the differences in daily behaviors such as exercise and diet across the patients and whether these factors could have vital effects on glycemic control.

## Introduction

Many studies have shown that diabetes mellitus not only results in specific complications but also leads to increased risks of cardiovascular disease and cancer [1-3]. Although it might have adverse outcomes, diabetes is now considered a chronic disorder that can usually be controlled with appropriate treatment, lifestyle management, and self-care measures to keep blood glucose in the target range [4,5].

Among self-care measures, the self-monitoring of blood glucose (SMBG) is a critical component for checking blood glucose levels [5-7]. Several studies have provided evidence that SMBG has notable benefits on glycemic control, and a recent meta-analysis showed that SMBG has beneficial effects on glucose control in both the short- and long-term [8-10]. Specifically, previous research articles have shown that SMBG is helpful for patients with diabetes mellitus type 1 in controlling blood sugar level. Furthermore, one randomized controlled trial (RCT) recruited patients with diabetes mellitus type 2 (DMT2) and observed them for at least 12 months, and the results suggested that SMBG improves diabetes control [11]. However, for patients with non–insulin-treated DMT2, the value of SMBG was inconsistent among the studies [11-15]. The difference may be due to the different research designs or targets of diabetes type in the studies.

To address the limitations of previous studies, we focused on patients with non–insulin-treated DMT2 who were receiving care from a single clinic and investigated the relationship between the frequency of SMBG and the patients’ glucose levels. In addition, there is growing evidence suggesting that digital technologies are being adopted as an additional method for health care systems to increase patient contact and enhance the effect of conventional care practices for diabetes patients [16,17]. We also focused on the patients who used a diabetes management app with self-care measures during the observation period. Therefore, the objective of this study was to analyze whether the app and SMBG affected glycemic control.

## Methods

### Data Collection

The Health2Sync mobile app and web-based Patient Management Platform were used to collect the data. These products were described in our previous study [16]. We collaborated with a large clinic focused on diabetes care in Taiwan that had been using these products. All the patients analyzed in this study belonged to the same diabetes clinic and received the clinic’s standard care. During a patient’s visit, the clinic’s health care professionals (HCPs) helped the patient register an account in the app and collected the patient’s demographic data, including gender, age, diabetes type, diabetes duration, and education level. After registration, smartphone-proficient users who were willing to use a digital management solution would start to use the Health2Sync mobile app; otherwise, patients would let HCPs sync their SMBG records from the blood glucose meters to their accounts during subsequent visits.

To assess the effects of SMBG and digital intervention from the Health2Sync mobile app, the patients’ SMBG records were averaged on a monthly basis, with the patients’ average in the first week after app registration designated as the baseline. Since each individual patient had different SMBG habits, only fasting blood glucose (FBG) records were included in the analyses for comparison. As the baseline blood glucose level of each patient was different, the maximum magnitude of the blood glucose increase or decrease could also be different, so we used the blood glucose percentage change instead of the blood glucose value change to assess the glycemic status improvement across the groups. The formula for that percentage change for each patient was *(mean of blood glucose value of each month – baseline blood glucose level) / baseline blood glucose level*.

### Patient Inclusion

The clinic had 6451 app-registered patients. To separate patients with different activities in the app, we defined 2 groups of users based on their frequency of using the Health2Sync mobile app. After registering for the app, the patients who used the app at least once a week on average were labeled as “app-engaged-users,” and those who used the app at most once a month on average were labeled as “only-data-uploaders,” as we believed that their SMBG were mainly uploaded by HCPs and they seldom used the app at home. The rest of the patients were excluded. At this stage, we excluded 2027 patients, leaving 1172 patients in the app-engaged-user group and 3252 patients in the only-data-uploader group.

**Figure 1:**
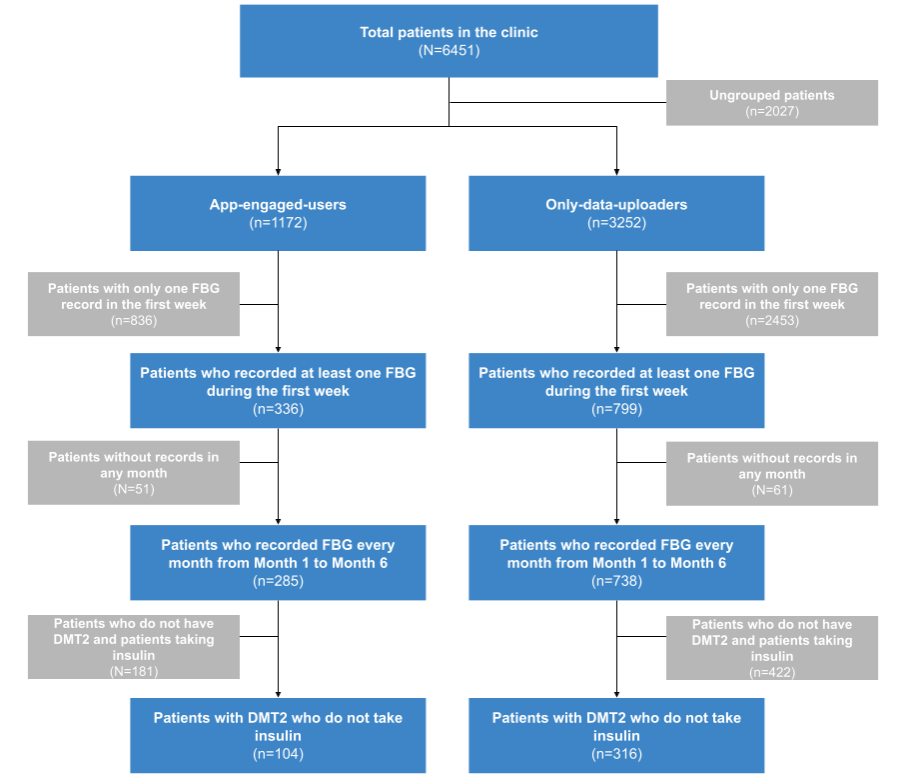
Inclusion flow chart of this study. DMT2: diabetes mellitus type 2; FBG: fasting blood glucose.

To calculate the patients’ blood glucose level change, we took the mean value of each patient’s FBG value recorded in the first week after app registration. Patients with only one FBG record in the first week were excluded as we believed this value was unrepresentative of the blood glucose level in the whole week. At this stage, we excluded 836 patients from the app-engaged-user group, with 336 remaining, and 2453 patients from the only-data-uploader group, with 799 remaining. The patients who had no record in any month from 1 to 6 months after app registration were also excluded, because a complete data set would be needed for later analyses, where blood glucose level is the dependent variable in modeling. At this stage, we excluded 51 and 61 patients from app-engaged-user and only-data-uploader groups, respectively. Finally, to eliminate the impact from differences due to diabetes type and medication, only DMT2 patients who do not take insulin treatments were included for the analyses. Eventually, we had 104 and 316 patients in the app-engaged-user and only-data-uploader groups, respectively. Figure 1 shows the inclusion flow chart described above.

In addition, we analyzed the blood glucose meters used by the patients. We were able to collect the blood glucose meter information for 320 patients. Among those, 302 patients used blood glucose meters that are compliant with the requirements for blood-glucose monitoring systems for self-testing in managing diabetes mellitus (ISO 15197:2013) [18], so we believe our study is based on accurate SMBG data (Multimedia Appendix 1).

### Analysis

#### Software and Model Used

We used R statistical software (version 3.6.1; R Foundation for Statistical Computing) [19] for all the statistical analyses, including *t* test (2-tailed), Pearson chi-square test, and one-way ANOVA. We also used the linear mixed model of the lme4 package for R (version 2015; Bates et al [20]).

#### Patient Characteristics

We used one-way ANOVA and Pearson chi-squared test for continuous and categorical variables, respectively, to check the homogeneity of the demographics across the 2 groups. In addition, we used one-way ANOVA to test whether the initial blood glucose levels of the 2 groups are different.

#### Statistical Modeling and Analysis

We started by creating a model (our original model) that included the key factors we believed would affect a patient’s blood glucose percentage change. We also wanted to include time (the nth month) as a factor for analysis, so a linear mixed model was used to analyze our original model. However, there were a few factors that were significantly different between the 2 patient groups. To confirm whether our model should include the interaction of these factors and the patient groups, we built a basic model that only included the patient groups and factors that were significantly different to check whether these factors had an effect on the blood glucose percentage change. Subsequently, for each of the significantly different factors, we built a new model based on the basic model that included the interaction of that individual factor with the groups. Finally, we built a new model that added all the factor-group interactions to the basic model to confirm whether the interactions of these factors and the groups have an effect on the blood glucose percentage change. We used the *P* value to determine whether these interaction factors should be added back into the original model [21].

### Ethical Considerations

Institutional Review Board approval was not sought for this study as it is based on retrospective analysis, and patients can freely choose whether or not to use the Health2Sync mobile app. The patients in both the app-engaged-user and only-data-uploader groups agreed to Health2Sync’s Privacy Policy before registering an account, giving H2 Inc the right to analyze their data for research purposes.

## Results

Table 1 presents the patients’ demographic characteristics stratified by the 2 groups. There were no significant differences in gender (*Χ*^2^_1_=0; *P*>.99), diabetes duration (t_418_=–0.69; *P*=.49), and the baseline blood glucose level (t_418_=–0.58; *P*=.56) between the 2 groups. However, significant differences were found in age (t_418_=–6.66; *P*<.001) and education level (*Χ*^2^_1_=45.44; *P*<.001).

**Table 1:** Patient characteristics analyses.

Characteristic			All patients (n=420)		App-engaged-user group (n=104)		Only-data-uploader group (n=316)	*P* value	
Age (years), mean (SD)			59.28 (11.29)		52.7 (12.06)		61.44 (10.16)	<.001	
**Gender, n (%)**									>.99
	Male	219 (52.1)		54 (51.9)		165 (52.2)			
	Female	201 (47.9)		50 (48.1)		151 (47.8)			
Diabetes duration (years), mean (SD)			9.23 (7.95)		8.74 (8.46)		9.39 (7.78)	.49	
**Education level, n (%)**									<.001
	Junior high school or lower	181 (46)		19 (20)		163 (54)			
	Senior high school or higher	212 (54)		75 (80)		137 (46)			
Initial blood glucose level (mg/dL), mean (SD)			135.62 (31.34)		134.16 (28.29)		136.10 (32.31)	.56	

Linear mixed modeling was used to estimate the effects from factors that could affect the patients’ blood glucose percentage change. In addition, due to the above analysis of patient characteristics, we know that there are significant differences in the patients’ age and education level between the patient groups. Therefore, we have to confirm whether the interactions of these factors and the groups should be put into the original model.

First, we built a basic model to check whether the patients’ group, age, and education level have an effect on the blood glucose percentage change. This basic model only included the patients’ group, age, and education level; these factors exhibited significant effects on the blood glucose percentage change (*P*<.001 for the patients’ age; *P*<.001 for the patients’ education level; *P*<.001 for the patients’ group). Second, we wanted to examine whether an interaction effect of the patients’ age and group has an effect on the blood glucose percentage change. We built a second model that included the same factors as the basic model, but also added an interaction effect of the patients’ age and group. The second model showed that the interaction effect of the patients’ age and group did not have a significant effect on the blood glucose percentage change (*P*=.53). We then built a third model that included the same factors as the basic model and the interaction effect of the patients’ education level and group. The third model showed that the interaction effect of the patients’ education level and group did not have a significant effect on the blood glucose percentage change (*P*=.48). Finally, we built a fourth model with the same factors as the basic model and added the 2 interaction effects—one for the patient’s age and group and another for the patient’s education level and group. In the fourth model, we found that the interaction effects did not have a statistically significant effect on the blood glucose percentage change (*P*=.109 for the interaction effect of the patients’ age and group; *P*=.94 for the interaction effect of the patients’ education level and group). Therefore, we decided not to incorporate the age-group and education level-group interaction factors into the original model.

After conducting the above analyses, our original model was kept, and the final, included variables consisted of the patients’ group, gender, diabetes duration, age, education level, the interaction effect of the nth month after registering an account, and the count of blood glucose measured in the nth month. Table 2 presents a summary of the new model.

**Table 2:** A summary of the new model.

Variable	Estimate	*t* test (*df*)	*P* value
Age	7.474 × 10^-4^	2.652 (1.71 × 10^4^)	.008
Education level^a^	2.927 × 10^-2^	4.996 (1.72 × 10^4^)	<.001
Patient groups^b^	–2.430 × 10^-2^	–3.873 (1.81 × 10^4^)	<.001
Gender^c^	2.753 × 10^-3^	0.534 (1.74 × 10^4^)	.59
Diabetes duration	1.010 × 10^-3^	2.823 (1.71 × 10^4^)	.005
Nth month	5.514 × 10^-3^	2.212 (8.26 × 10^2^)	.003
Count of blood glucose measured	1.403 × 10^-4^	1.611 (2.37 × 10^4^)	.11
Count of blood glucose measured in the nth month	–3.352 × 10^-4^	–8.266 (1.97 × 10^4^)	<.001

^a^The group of patients with an education level of junior high school or lower was set as the baseline.

^b^The only-data-uploader group was set as the baseline.

^c^The male patient cohort was set as the baseline.

**Figure 2:**
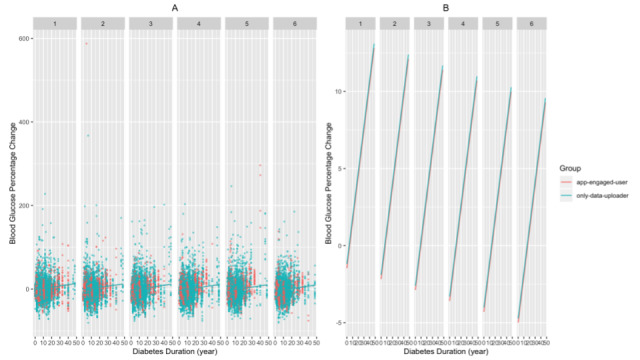
The relationship between blood glucose percentage changes and diabetes duration for each month as (A) a jittered scatter plot and (B) regression lines. In (A), the count of blood glucose measured in the nth month and the patients’ age and educational level are fixed, and the overlaid regression lines are based on the estimated coefficients from the mixed model.

**Figure 3:**
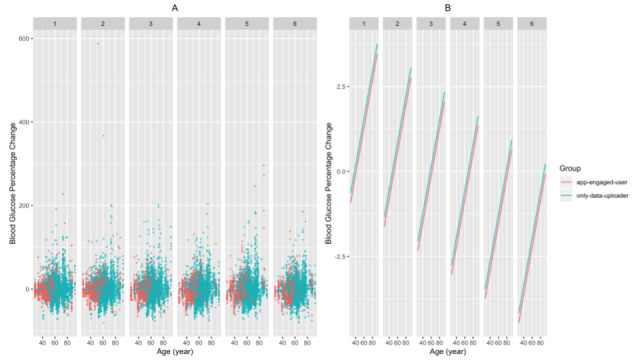
The relationship between blood glucose percentage changes and patient age for each month as (A) a jittered scatter plot and (B) regression lines. In (A), the count of blood glucose measured in the nth month and the patients’ diabetes duration and educational level are fixed, and the overlaid regression lines are based on the estimated coefficients from the mixed model.

**Figure 4:**
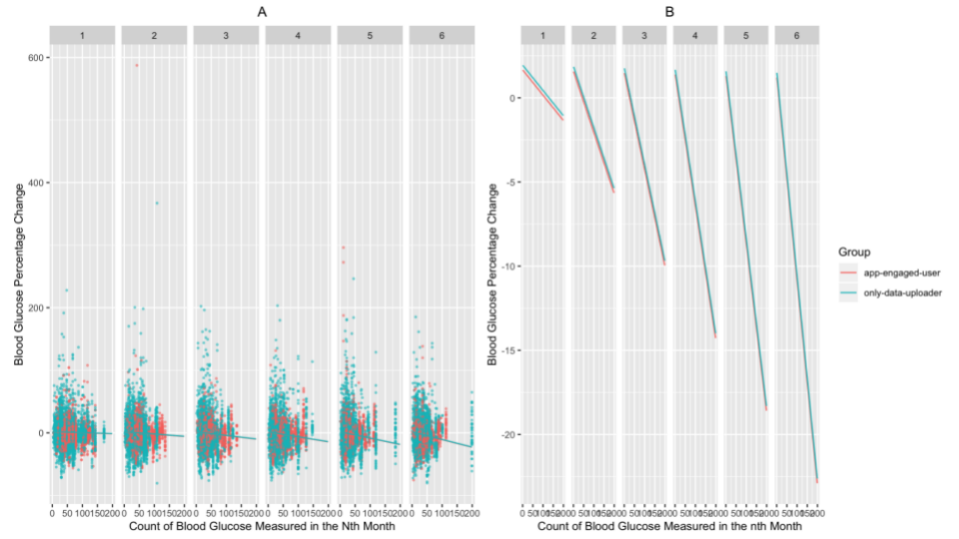
The relationship between blood glucose percentage changes and the count of blood glucose measured in each month as (A) a jittered scatter plot and (B) regression lines. In (A), the diabetes duration is fixed, and the overlaid regression lines are based on the estimated coefficients from the mixed model.

**Figure 5:**
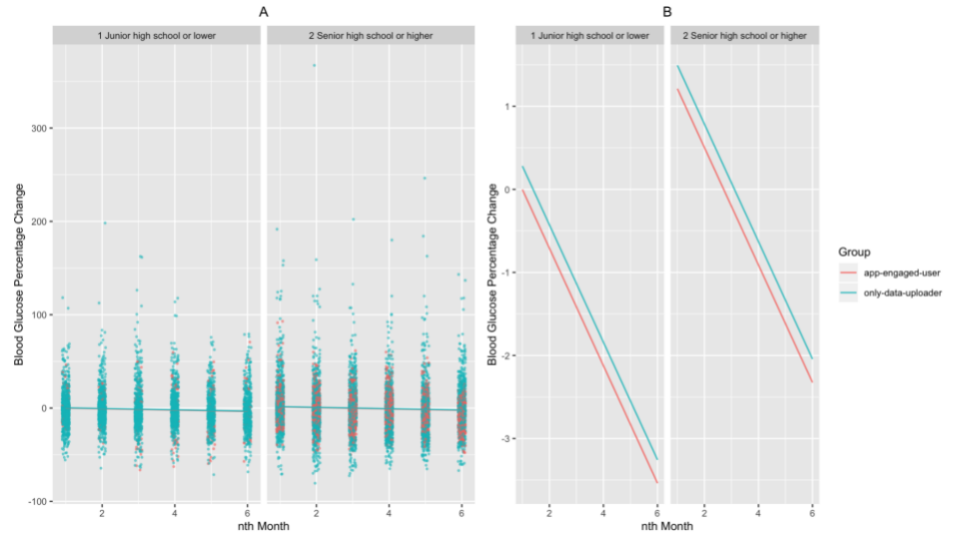
The relationship between blood glucose percentage changes and educational level for each month as (A) a jittered scatter plot and (B) regression lines. In (A), the count of blood glucose measured in the nth month and the patients’ diabetes duration and age are fixed, and the overlaid regression lines are based on the estimated coefficients from the mixed model.

We found that the app-engaged-user group had significantly greater decreases in blood glucose percentage change than the only-data-uploader group (β estimate=–2.430 × 10^-2^; *t*=–3.873, *df*=1.81 × 10^4^; *P*<.001 for the patients of the app-engaged-user group). In addition, for patients with shorter diabetes duration and those who are younger, the magnitudes of the drops in blood glucose percentage change were more profound (β estimate=1.010 × 10^-3^; *t*=2.823, *df*=1.71 × 10^4^; *P*=.005 for diabetes duration; β estimate=7.474 × 10^-4^; *t*=2.652, *df*=1.71 × 10^4^; *P*=.008 for the age of the patients; Figures 2-3). We also found that the frequency of SMBG enlarged the decreases in blood glucose along the interaction months (β estimate=–3.352 × 10^-4^; *t*=–8.266, *df*=1.97 × 10^4^; *P*<.001 for the nth month × the count of blood glucose in the nth month; Figure 4). Additionally, when the group of patients with an education level of junior high school or lower was set as the baseline, these patients had significantly greater decreases in blood glucose percentage change than those with an education level of senior high school or higher (β estimate=2.927 × 10^-2^; *t*=4.996, *df*=1.72 × 10^4^; *P*<.001 for patients with an education level of senior high school or higher; Figure 5). Lastly, the gender of the patients did not significantly affect the percentage change (β estimate=2.753 × 10^-3^; *t*=0.534, *df*=1.74 × 10^4^; *P*=.59 for female patients, with male patients as the baseline).

## Discussion

Our study was based at a single clinic to minimize differences between the frequency of patient visits, level of health education, and quality of care received for the app-engaged-user and only-data-uploader groups. Our results showed that there were 6 significant factors—the patients’ group (app-engaged-user or only-data-uploader), age, diabetes duration, education level, gender, and the count of blood glucose measured in the nth month—that were more strongly associated with changes in the patients’ blood glucose. We found that patients who are app-engaged, younger, and less-educated and have shorter diabetes duration saw a steeper decrease in their blood glucose percentage change. We also found that the interaction between the nth month of recording SMBG and the SMBG count of that month affected blood glucose level significantly. Therefore, this interaction deserves more attention than total SMBG count. However, we found that the gender of the patients did not significantly affect the percentage change.

As previously mentioned, in many studies for patients with non–insulin-treated DMT2, the value of SMBG is inconsistent [8,11,22-24]. Some studies have demonstrated that SMBG was effective in controlling blood glucose [8,22,23,25], whereas other studies claimed that SMBG was not effective [12,26]. These inconsistencies are mainly due to differences in the trial designs, populations studied, and outcome indicators. However, in our findings, we used the count of blood glucose measured in the same month instead of the use of SMBG as a measurement. This is different from some previous studies. Diabetes patients test SMBG differently according to their current blood glucose status. Generally, when a patient’s blood glucose becomes more stable, the count of SMBG will decrease. Therefore, it is more accurate to look at how the month of SMBG testing and count of SMBG each month affects blood glucose levels than total SMBG count.

Patients with longer diabetes duration may be affected by more diabetes symptoms [27-29], so their control of diabetes is usually worse than patients with a shorter diabetes duration. In addition, aging is associated with physiological changes that may lead to systemic alterations [30]. These systemic alterations may affect mental and physical functioning, increasing the chances of morbidity, multimorbidity, and mortality [30]. Older patients with diabetes may have macrovascular and microvascular complications and geriatric syndromes [28,31], so their control of diabetes is usually worse than younger patients with diabetes.

A common assumption is that patients with higher educational levels would have more knowledge about diseases and therapies, and thus, they would be able to better comply with therapies. However, previous studies have found that even highly educated patients may not sufficiently understand their conditions or truly believe in the benefits of therapy compliance, whereas patients with lower education levels may trust the doctor’s advice more and exhibit better compliance [32,33]. This could explain our finding that the blood glucose percentage drop of the patients with an education level of junior high school or lower was greater than those with an education level of senior high school or higher. In addition, our study showed that the blood glucose percentage decrease of the patients who used the Health2Sync mobile app was more than those who did not use the app. For patients with the same education level, those using the Health2Sync mobile app had a greater decrease in blood glucose percentage than the patients who did not use it. Furthermore, regardless of the level of education, the patients who used the Health2Sync app experienced a larger drop in blood glucose levels than those who do not use it. The Health2Sync app benefits users because it allows them to record their daily behaviors together with blood glucose readings, and the app has a bot that provides automated analyses, alerts, encouragements, and personalized educational content [16]. For the app-engaged-user group, 66% (69/104) of the patients recorded behavioral factors in addition to self-reported outcomes, most commonly entering diet, and 85% (88/104) viewed at least one educational content or interactive educational guide the app provided. Previous studies have shown that patient education and diet management are crucial for improving blood glucose [8,23,25,27]. There is growing evidence suggesting that gender affects the pathophysiology of many diseases, but in our study, gender did not significantly affect blood glucose percentage change [34-37]. Our study focused on a single clinic with limited samples; future studies should consider including a few more clinics to obtain more data samples for analyses. The other limitation is that we did not consider the differences in daily behaviors such as exercise and diet across the patients, and that these factors could have vital impacts on glycemic control. Future studies should also include these behaviors for analyses.

Diabetes is approaching epidemic proportions globally, and it places an enormous burden upon both the patients and countries’ health systems. It is especially difficult for low- and middle-income countries, due to insufficient equipment and clinics, to cope with the rise in diabetes and other chronic diseases [25,38]. The Health2Sync app can enhance the care for patients with diabetes and solve resource-limited problems.

Additionally, our study showed positive results at a single diabetes management clinic using real-world data without prior RCT settings. RCTs are generally considered by regulators to be the gold standard for establishing the causal relationship between medication and patient outcomes, but it is incapable of reflecting real clinical practice in which heterogeneous scenarios exist [39-41]. As digital interventions are to be applied to all patients, we believe that our study with real-world data is more convincing in demonstrating efficacy.

In conclusion, through the retrospective analyses, we showed that the Health2Sync app and SMBG contribute to the improvement and control of blood glucose. Further studies are needed to reveal whether different clinical care methods have an impact on diabetes treatment.

## Appendix

Multimedia Appendix 1 List of blood glucose meters used by patients.

## Abbreviations

DMT2: diabetes mellitus type 2
FBG: fasting blood glucose
HCP: health care professional
RCT: randomized controlled trial
SMBG: self-monitoring of blood glucose
