# Supplementary material for: Real-world Benefits of Diabetes Management App Use and Self-monitoring of Blood Glucose on Glycemic Control: Retrospective Analyses
Source: JMIR Mhealth Uhealth. 2022 Jun 15;10(6):e31764. doi: 10.2196/31764 (PMC9244648; doi:10.2196/31764)
Supplement: Multimedia Appendix 1 [file mhealth_v10i6e31764_app1.docx]

**Multimedia Appendix 1**. List of blood glucose meters used by patients.

| Brand | Model | Number of patients |
| --- | --- | --- |
| ADC | Contour Plus | 10 |
| Abbott | Freedom Lite | 4 |
| Abbott | Lite | 2 |
| BIONIME | GM700S | 145 |
| BIONIME | GM550 | 10 |
| FORA | GD40b | 1 |
| General Electric | GE100 | 8 |
| OneTouch | Ultra Easy | 62 |
| OneTouch | Ultra Plus Flex | 1 |
| Roche | Performa | 178 |
| Roche | Guide | 66 |
| Roche | Active | 1 |
| Roche | Instant | 1 |
